# Longitudinal Outcomes of Left Ventricular Outflow Tract Obstruction in Aortic Stenosis Versus Hypertrophic Obstructive Cardiomyopathy

**DOI:** 10.3390/medicina61060971

**Published:** 2025-05-23

**Authors:** Joy Yi-Shan Ong, Tony Yi-Wei Li, Aloysius Sheng-Ting Leow, Swee-Chye Quek, William Kok-Fai Kong, Weiqin Lin, Ping Chai, Tiong-Cheng Yeo, Raymond Ching-Chiew Wong, Ching-Hui Sia, Kian-Keong Poh

**Affiliations:** 1 Department of Cardiology, National University Heart Centre Singapore, 5 Lower Kent Ridge Road, Singapore 119074, Singapore; joy.ong@mohh.com.sg (J.Y.-S.O.);; 2 Department of Medicine, Yong Loo Lin School of Medicine, National University of Singapore, Level 11, NUHS Tower Block, 1E Kent Ridge Road, Singapore 119228, Singapore

**Keywords:** LVOT obstruction, AS, HOCM, heart failure, mortality outcomes

## Abstract

*Background and Objectives:* Aortic stenosis (AS) and hypertrophic obstructive cardiomyopathy (HOCM) are two disease entities that result in left ventricular outflow tract (LVOT) obstruction. We sought to evaluate the longitudinal outcomes of fixed obstruction in severe valvular AS versus dynamic flow obstruction in HOCM. *Materials and Methods*: Consecutive data with index echocardiographic diagnoses of severe AS and HOCM were collected in a tertiary academic centre between 2010 and 2017. Demographics, comorbidities and clinical outcomes were compared. *Results*: A total of 134 patients were studied. In the AS group, the mean MPG was 57.2 mmHg ± 13.9, the mean AVA was 0.7 cm^2^ ± 0.2, and the mean Vmax was 4.7 m/s ± 0.5 (*p* < 0.001). In the HOCM group, the mean LVOT gradient was 60.1 mmHg ± 35.5, the mean IVSd was 17.5 mm ± 4.6, and the mean LVPWd was 12.9 mm ± 2.9 (*p* < 0.001). Kaplan–Meier curves showed lower cumulative survival with an early separation in heart failure outcomes in the AS arm compared with the HOCM arm (*p* = 0.023). Similarly, there were higher rates of all-cause mortality for AS compared with HOCM (*p* = 0.001). For the multivariable Cox regression analysis, AS was significantly associated with a higher incidence of heart failure compared with HOCM after adjusting for the baseline demographics, comorbidities and echocardiographic parameters. There were no significant differences in terms of stroke or cardiovascular (CV) hospitalisation outcomes between the two cohorts. *Conclusions*: Fixed LVOT obstruction in AS was associated with worse outcomes of heart failure and all-cause mortality compared with dynamic LVOT obstruction in HOCM. Severe AS was an independent predictor of heart failure outcomes after adjustments.

## 1. Introduction

Left ventricular outflow tract (LVOT) obstruction encompasses a heterogenous group of conditions that may be familial or acquired. They include fixed anatomic obstructions at the valvular, subvalvular and supravalvular levels in aortic stenosis (AS), as well as dynamic LVOT obstruction in hypertrophic cardiomyopathy (HCM). Obstructions may be present at any phase within the cardiac cycle, from peri-systole in AS to late systolic culmination in obstructive HCM (HOCM). Patients may present with a spectrum of symptoms from angina to dizziness, syncope, heart failure, arrhythmias or sudden cardiac death [1,2].

AS is a restricted left-sided valvular opening secondary to leaflet calcification and fibrosis, stemming from a myriad of congenital and acquired aetiologies [3,4]. AS can be classified into mild, moderate and severe severities according to the mean pressure gradient (MPG) (≥40 mmHg for severe, 20–40 mmHg for moderate and <20 mmHg for mild), aortic valve peak velocity (Vmax) (≥4.0 m/s for severe, 3.0–4.0 m/s for moderate and 2.6–2.9 m/s for mild) and aortic valve area (AVA) (<1.0 cm^2^ for severe, 1.0–1.5 cm^2^ for moderate and >1.5 cm^2^ for mild) [5,6]. On the other hand, HCM is the most common genetic cardiomyopathy of autosomal dominance inheritance [1]. It is morphologically characterised by marked left ventricular hypertrophy, in the absence of abnormal loading conditions and systemic processes [1]. It is defined by cardiac imaging dimensions of a maximal end-diastolic left ventriclular wall thickness ≥15 mm in adults and a z score ≥2 standard deviations above the mean, adjusted for the body surface area in the presence of a family history in children [1]. Obstruction in HCM is defined as the presence of an instantaneous peak LVOT gradient ≥30 mmHg either at rest or with provocation [1]. The pathobiology of hypertrophic cardiomyopathy is primarily derived from dynamic LVOT obstruction due to a mixed interplay of systolic anterior motion (SAM) of the mitral valve leaflets when mitral leaflets encroach into the LVOT, with flow acceleration during systole causing a drag effect, augmented by hyperdynamic circulation with obliteration of the left ventricular cavity in systole, asymmetrical septal hypertrophy, and a steep LV inflow-to-outflow (aorto-LVOT) angle at various levels depending on the combination of factors [1,7,8]. Coupled with phenotypic manifestations of elongated mitral valve leaflets, along with submitral valve apparatus abnormalities such as anomalous and/or apically displaced papillary muscle insertion, dynamic LVOT obstruction occurs [1,8]. There is dynamic reactivity to preload, afterload and contractility in HCM [1].

In phenotypically distinct ways, AS and HCM result in haemodynamically significant left ventricular outflow tract obstruction (LVOTO). Structurally, in view of an impedance to forward flow, both conditions result in high afterload, a reduced stroke volume, decreased cardiac output and heart failure [2]. Left ventricular remodelling in response to increased intracavitary pressure and wall stress leads to structural alterations of concentric hypertrophy, dilatation and dysfunction [2]. They can occur in isolation as part of separate illness processes or coexist in tandem as part of the same disease complex, posing management and prognostic challenges. An increasing spotlight has been cast on the presentation, diagnosis and management of LVOTO in different clinical scenarios. However, little is known about the outcome differences between fixed obstruction peri-systole peaking mid-systole in severe AS and dynamic flow obstruction culminating in mid-to-late systole in obstructive HCM (HOCM), with an absence of head-to-head studies comparing the two disease entities [9]. In this study, we sought to evaluate the clinical characteristics and longitudinal outcomes between valvular and subaortic LVOT gradients in an Asian cohort.

## 2. Methods

### 2.1. Study Design and Population

This was a retrospective cohort study comparing consecutive patients diagnosed with severe AS from September 2011 to December 2015 and obstructive HCM from August 2010 to October 2017 via echocardiography at a tertiary academic hospital in Asia. A comparable mean pressure gradient (MPG) was used for pressure gradient quantification in AS against the left ventricular outflow tract (LVOT) gradient at rest for pressure gradient assessment in HCM in our study, as discussed below. Ethics approval was obtained from the local institutional review board, the National Healthcare Group (NHG) Domain Specific Review Board (DSRB). For patients with multiple echocardiographic studies performed during the study period, only the index echocardiography was used for diagnosis of severe AS or HOCM. Grading of the severity of AS, diagnosis of HOCM and evaluation of echocardiographic parameters was performed in accordance with the European Association of Cardiovascular Imaging guidelines and the American Society of Echocardiography [1,5,10]. The aortic valve area was calculated using the continuity equation, and patients with classical and paradoxical low-flow low-gradient AS as well as traditional high-gradient AS were included in the study. Relevant data such as demographics, comorbidities, echocardiographic parameters, treatments and outcomes were collected from the electronic medical records and then compared between severe AS and HOCM cases.

### 2.2. Study Endpoints and Statistical Analysis

The primary study endpoint was all-cause mortality, while secondary endpoints included subsequent heart failure, stroke and cardiovascular hospitalisation. We presented categorical variables as frequencies and percentages, continuous variables as means ± standard deviations and used a chi-square test or independent samples *t*-test for comparison, respectively. The Kaplan–Meier estimate was used for the outcome of all-cause mortality, while the cumulative incidence function estimates were used for the secondary endpoints of subsequent heart failure, stroke and cardiovascular hospitalisation to account for the competing risk of all-cause mortality. The differences were evaluated with a log-rank test [11]. Similarly, the Cox proportional hazards regression model was employed for all-cause mortality, while the Fine and Gray competing risks (for all-cause mortality) regression model was employed for the secondary endpoints, which were presented as a hazard ratio (HR), 95% confidence interval (95% CI) and *p* value [12]. The multivariable regression models were adjusted for age, female sex, body mass index (BMI), chronic kidney disease, ischaemic heart disease and pressure gradient (aortic valve [AV] mean pressure gradient for AS and left ventricular outflow tract [LVOT] gradient for HOCM). All *p* values less than 0.05 were considered statistically significant. Statistical analyses were performed using R Statistical Software (v4.4.1; R Core Team, Vienna, Austria, 2023) and Rstudio (v2024.9.0; Rstudio Team, Boston, MA, USA, 2024) with the following key packages: ggsurvfit (v1.0.0, Sjoberg, Rennesøy, Norway, 2024) and tidycmprsk (v1.0.0, Sjoberg, 2023).

## 3. Results

A total of 85 severe AS and 49 obstructive HCM patients were included in this study (Table 1). Compared with the severe AS patients, the HOCM patients were younger (64.8 ± 16.6 years versus 71.3 ± 13.5 years, *p* = 0.015), had higher BMI values (26.0 ± 5.4 kg/m^2^ versus 23.8 ± 4.2 kg/m^2^, *p* = 0.010) and less likely to have chronic kidney disease (CKD) (2.0% versus 64.7%, *p* < 0.001). There were no significant differences in terms of sex distribution (*p* = 0.858), ethnicity (*p* = 0.475) or other comorbidities between the two cohorts.

The echocardiographic parameters are reported in Supplementary Table S1. In the severe AS cohort, the mean pressure gradient (MPG) was 57.2 ± 13.9 mmHg (*p* < 0.001), the peak aortic jet velocity (Vmax) was 4.7 ± 0.5 m/s (*p* < 0.001), and the mean aortic valve area (AVA) was 0.7 ± 0.2 cm^2^ (*p* <0.001). In the HOCM cohort, the left ventricular outflow tract (LVOT) gradient was 60.1 ± 35.5 mmHg (*p* < 0.001), the LVOT Vmax was 3.8 ± 0.9 m/s (*p* < 0.001), the interventricular septum diameter in diastole (IVSd) was 17.5 ± 4.6 mm (*p* < 0.001), and the left ventricular posterior wall thickness in diastole (LVPWd) was 12.9 ± 2.9 (*p* = 0.008). Systolic anterior motion of the mitral valve was observed in 57.1% (*n* = 28) of the HOCM patients. When comparing the two cohorts, the HOCM patients had higher left ventricular ejection fraction (LVEF) rates (69.0 ± 7.0% versus 53.8 ± 13.7%, *p* < 0.001) but similar rates of pulmonary artery systolic pressure (PASP) (35.1 ± 10.2 mmHg versus 39.1 ± 15.3 mmHg, *p* = 0.112) and prevalence of left ventricular hypertrophy (LVH) (77.6% versus 76.5%, *p* = 0.982). The AS patients had a higher stroke volume (68.6 ± 21.6 mL versus 51.2 ± 29.3 mL, *p* < 0.001) and cardiac output (4.8 ± 1.6 L/min versus 3.5 ± 2.2 L/min, *p* < 0.001) compared with the HOCM patients. There was no significant difference in terms of diastology between the two cohorts (*p* = 0.101).

The HOCM patients were more likely to be on beta-blockers than the severe AS patients (69.4% versus 40.0%, *p* = 0.001) but were similar in terms of antithrombotic agents, statins (*p* = 0.983), angiotensin converting enzyme inhibitors (ACEis) and angiotensin receptor blockers (ARBs) (*p* = 0.382) (Table 1). A minority of the severe AS patients underwent either surgical or transcatheter aortic valve replacement (AVR) (37.6%, *n* = 32), with a mean duration of 1.9 ± 1.7 years to replacement. Only 8.2% (*n* = 4) of the HOCM patients underwent implantable cardioverter defibrillator (ICD) implantation, based on the ACC consensus criteria of unexplained syncope in two patients and non-sustained ventricular tachycardia (NSVT) under monitoring in two patients. None of the HOCM patients underwent alcohol septal ablation or surgical myectomy. Mavacamten had not been available for use at the time of the study.

**Figure 1:**
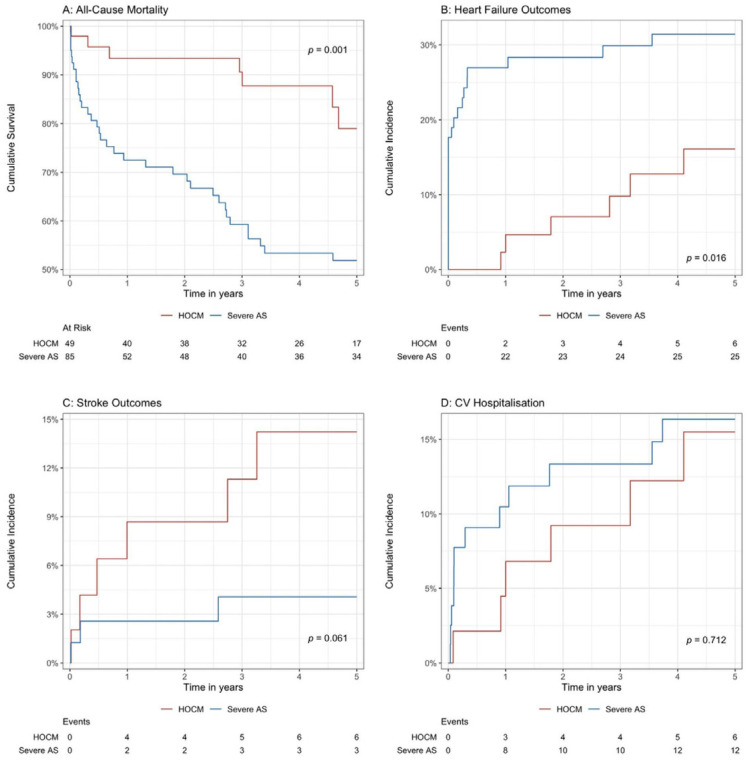
(**A**–**D**) Kaplan–Meier and cumulative incidence function estimates of outcomes comparing severe AS and HOCM patients.

The overall cohort was followed up for a mean duration of 2.9 ± 2.1 years, with the HOCM patients having a longer follow-up duration (3.4 ± 1.8 years versus 2.7 ± 2.2 years, *p* = 0.042). The HOCM patients had significantly lower incidence rates for subsequent heart failure (12.2% versus 29.4%, *p* = 0.023) and all-cause mortality (14.3% versus 41.2%, *p* = 0.001) (Table 1). Similar results were seen in the Kaplan–Meier and cumulative incidence function estimates– with higher rates of all-cause mortality (*p* = 0.001) and subsequent heart failure outcomes (*p* = 0.016) seen in the severe AS patients compared with the HOCM patients (Figure 1A,B). There were no significant differences in terms of rate of stroke (*p* = 0.061) or CV hospitalisation outcomes (*p* = 0.710) (Figure 1C,D).

Regarding the Cox proportional hazards time-to-event analysis (Table 2), severe AS was associated with higher all-cause mortality rates (HR 3.54, 95% CI 1.57–7.98, *p* < 0.001) compared with the HOCM patients on univariate analysis but was not significant under multivariable analysis (aHR 1.44, 95% CI 0.50–4.16, *p* = 0.504) after adjusting for the age, female sex, BMI, comorbidities, and pressure gradient. Further subgroup analysis of the Kaplan–Meier curve (Supplementary Figure S1) revealed that the severe AS patients with chronic kidney disease had significantly higher rates of all-cause mortality (*p* < 0.001), while the severe AS patients without chronic kidney disease had similar survival rates to the HOCM patients.

For the outcome of subsequent heart failure, severe AS was an independent risk factor (aHR 3.66, 95% CI 1.43–9.38, *p* = 0.007) compared with HOCM under multivariable Fine and Gray competing risks regression analysis, even after adjusting for the same co-variates. Severe AS was not associated with stroke (HR 0.43, 95% CI 0.05–3.61, *p* = 0.440) or CV hospitalisation outcomes (HR 0.81, 95% CI 0.15–4.40, *p* = 0.811). The full multivariable regression models are reported in Supplementary Table S2.

## 4. Discussion

The findings in our cohort suggest that the AS patients were more elderly and had lower body mass indexes and higher incidence rates of multimorbidity which was most significant for CKD compared with the HOCM patients. LVOT obstruction was comparable between the severe AS and obstructive HCM patients in our cohort at an MPG of 57.2 ± 13.9 mmHg and LVOT gradient of 60.1 ± 35.5 mmHg, respectively. A minority in both arms underwent intervention (AVR for AS and ICD implantation for HOCM). More HOCM patients were on beta blockers compared with AS patients. More HOCM patients were followed up with for a longer duration than AS patients and had lower incidence rates of heart failure and all-cause mortality compared with the AS patients. Severe AS was independently associated with worse heart failure outcomes when compared with HOCM.

AS poses a relatively uniform degree of obstruction present in peri-systole, while in HCM, obstruction rises rapidly from minimal in early systole to its peak in late systole in a “spike and dome” pattern [13]. Thus, Geske et al. recommended the use of a mean gradient, or the average of instantaneous gradients across systole in the application of LVOT assessment and severity in AS, compared to the peak instantaneous gradient, which correlates well with the peak instantaneous gradient in HCM [13]. As such, the MPG was used for pressure gradient quantification in AS and LVOT gradients for pressure gradient assessment in HCM in our study.

Epidemiologically, the prevalence of AS increases exponentially with age, occurring in 0.2% of those aged 50–59, 1.3% of those aged 60–69, 3.9% of those aged 70–79 and 9.8% of those aged 80–89 [14]. It occurs from lipid buildup, inflammation and calcification, accelerating leaflet calcification and fibrosis to culminate in decreased leaflet mobility, restricted opening and left ventricular outflow tract impediment [15]. It has a shared pathophysiology attributed to accelerated atherosclerotic deposition similar to chronic cardiovascular diseases such as coronary artery disease (CAD) and chronic kidney disease (CKD) [15]. In the absence of congenital abnormalities, cardiovascular risk factors and multimorbidity, such as hypertension, coronary artery disease, chronic kidney disease and frailty, are present with higher incidence rates in this more elderly cohort [16]. On the contrary, the estimated prevalence of HCM is 0.2% globally, with the highest presentation rate being in the third decade of life, although it can present at any age group [17,18]. It is the most common cause of sudden cardiac death in athletes under 35 [17,18]. Obstructive HCM (HOCM) occurs in one third of HCM patients [18]. 60% of patients with HCM possess a sarcomeric genetic mutation of MYH7 and MYBPC3 most commonly associated with an autosomal dominant Mendelian inheritance with incomplete penetrance and variable expression [17]. Molecularly, large cardiomyocytes resulting in myocardial disorganisation disrupt the regular architecture and intercellular junction, resulting in clinical manifestations of fibrosis that can cause asymmetrical hypertrophy, impaired filling, microvascular dysfunction, arrhythmogenicity and subsequent dynamic left ventricular outflow tract hindrance [19]. Functional effects impairing various intermediary components of the acto-myosin cross-bridge cycling result in inefficient force and tension generation [19]. In this way, the demographics of the HOCM population tended to be younger with less comorbidities compared with the severe AS population.

Cardiac damage staging in AS takes into account extra-aortic lesions, haemodynamic indices and function, including the left ventricular mass, filling pressures, systolic function, mitral and tricuspid valves and left atrial, pulmonary and right ventricular function. On top of the existing aortic valve lesion, the model helps predict heart failure, adverse cardiac events and mortality in AS, where more advanced stages are associated with worse outcomes [20,21,22]. One study displayed that asymptomatic patients with moderate AS had similar poor all-cause mortality rates to severe AS within the same cardiac damage stage [22]. In AS, adverse left ventricular myocardial remodelling, fibrosis and hypertrophy secondary to chronic pressure overload effects can lead to elevated filling pressures, high left atrial pressures, pulmonary capillary engorgement and resultant pulmonary oedema [22,23]. Among AS patients, 5–10% have low EFs and an absence of contractile reserves in a classical low-flow low-gradient state, which can lead to a low forward stroke volume and a backlog into the pulmonary vasculature [24,25]. Classical low-flow low-gradient AS has been shown to have poor heart failure and mortality outcomes pre- and post-intervention compared with its paradoxical low-flow low-gradient AS and high-gradient AS counterparts [24,25]. In HCM, there is typically phenotypic preservation of the ejection fraction with little ventricular remodelling and fibrosis [26]. Heart failure presentation in the HCM cohort was characterised by impaired forward cardiac output and elevated left ventricular filling. The elevated left ventricular end diastolic pressure (LVEDP) from associated small and stiff chambers lead to increased left atrial pressure and pulmonary artery pressure [26]. Primary pulmonary hypertension has also been described in HCM patients with normal pulmonary artery wedge pressure [26]. Hence, the multifaceted extra-valvular sequelae of cardiac damage in AS portends worse outcomes for heart failure outcomes compared with a relatively intact cardiac ecosystem in obstructive HCM.

The leading cause of cardiac death in AS patients is heart failure followed by sudden death in our cohort, similar to the CURRENT-AS registry [27]. In HCM, the leading causes of mortality are ischemic heart disease (IHD) at 8.45%, heart failure at 0.53%, hypertensive diseases at 2.41% and cerebrovascular diseases at 1.45% [28]. Age above 75 is a significant factor in mortality determination in HCM [28]. Mortality in untreated AS is poor and directly correlates to disease severity [29]. Untreated AS diagnoses of mild, moderate, or severe gradings are associated with 25.0% (95% CI: 23.8–26.1%), 33.5% (95% CI: 31.0–35.8%) and 44.9% (95% CI: 39.9–49.6%) 4-year all-cause mortality rates in a real-world 1-million large registry in the United States (US) [29]. There is no available pharmacotherapy to slow progression or improve the mortality rates of AS. Only aortic valve intervention has been shown to be a significant risk modifier of AS survival rates and outcomes via direct relief of left ventricular outflow obstructions [3], with transcatheter techniques being shown to be non-inferior to surgical techniques across all risk profiles [30,31,32,33,34]. The early mortality rate within 30 days after surgical aortic valve replacement (SAVR) is 5.6%, with the relative survival rates (excluding early deaths) after 5, 10 and 15 years being 94.6%, 84.7% and 74.9%, respectively [35], while the relative survival rates of the transcatheter aortic valve replacement (TAVR) cohort were 95.4%, 90.2% and 83.8% at 30 days, 1 year and 3 years, respectively [36]. In AS, the mean pressure gradient has been shown to be a strong predictor of outcomes after AVR, with superior outcomes in patients with more elevated gradients [3]. Although only a minority of the AS patients in our study cohort underwent AVR (36.7%, *n* = 32), procedural advancements such as commissural alignment during transcatheter AVR are worth highlighting, as it has been shown to improve coronary access, valve hemodynamics and durability, especially when guided by pre-procedural computed tomography imaging [37,38]. In HCM, therapeutic modifiers such as alcohol septal ablation, surgical myectomy and heart transplantation have been shown to significantly improve long-term morbidity and mortality over the past two decades [39]. HCM-related mortality has reduced steadily in the United States from the early 2000s to 2019, with the 5- and 10-year mortality rates of HCM in adults being comparable to the general US population [39]. Septal reduction therapies (SRTs) are offered to patients with LVOTO exhibiting medication-resistant symptoms in experienced centres, and ICD placement is recommended for HOCM patients with prior cardiac arrest, sustained ventricular tachyarrhythmia or major risk factors for sudden cardiac death [40]. Given the progressive nature of valvular damage in a frailer subset of patients compared with a hyperdynamic and more robust disease complex in a younger subset of patients, the mortality rate is worse in the former compared with the latter, with surgery targeting LVOT obstructions and gradients in both groups resulting in excellent outcomes in the former and an improved outcome, to a lesser extent, in the latter.

Non-vasodilating betablockers are recommended with a class I recommendation within the HCM guidelines [1]. Sympathetic alteration of betablockers reduce ionotropy, chronotropy and blood pressure, thereby reducing myocardial workload and demand. Beta-adrenergic receptor blockade improves dynamic outflow tract obstruction by decreasing the maximal contraction velocity, which works to reduce the degree of systolic anterior motion of the mitral valve [40]. It also lengthens the diastolic phase, allowing coronary perfusion on top of control of arrhythmias and angina [28]. On the contrary, beta blockers have historically been used with caution in patients with AS in the absence of other indications, in light of fear of left ventricular depression and worse afterload mismatch in the context of fixed left ventricular outflow tract obstruction [22,41,42]. However, in recent years, there has been increasing research suggesting safety and improvements in mortality outcomes with concomitant use of beta blockers in AS [22,41,42].

To the best of our knowledge, this was the only outcome study to compare left ventricular outflow tract obstruction in severe aortic stenosis and obstructive hypertrophic cardiomyopathy in Asia at the time this manuscript was written. A limitation of this study is it was a retrospective observational study from a single tertiary centre, with inherent risks of selection bias and a relatively small sample size that cannot be generalised to all AS and HCM populations. Significant differences existed between the baseline characteristics of the included AS and HCM patient populations. These findings were not unexpected, given the known demographic differences between these disease processes.

## 5. Conclusions

Fixed LVOT obstruction in severe AS was associated with worse outcomes of heart failure and all-cause mortality compared with dynamic LVOT obstruction in obstructive HCM. There were no significant differences in terms of stroke or CV hospitalisation outcomes between the two cohorts. Severe AS was an independent predictor of heart failure outcomes after adjustments.

## Figures and Tables

**Table 1:** Clinical characteristics comparing severe AS and HOCM patients.

Variables	N	Overall N = 134	Severe AS N = 85	HOCM N = 49	*p* Value
Baseline Characteristics					
Age (years), mean (SD)	134	68.9 (15.0)	71.3 (13.5)	64.8 (16.6)	0.015
Female sex, *n* (%)		67 (50.0)	43 (50.6)	24 (49.0)	0.858
Ethnicity, *n* (%)					0.475
Chinese		85 (63.4)	56 (65.9)	29 (59.2)	
Malay		23 (17.2)	12 (14.1)	11 (22.4)	
Indian		11 (8.2)	6 (7.1)	5 (10.2)	
Others		15 (11.2)	11 (12.9)	4 (8.2)	
Height (cm), mean (SD)		159 (10)	159 (10)	160 (10)	0.394
Weight (kg), mean (SD)		63 (15)	60.0 (13.4)	67 (17)	0.012
BSA (m^2^), mean (SD)		1.6 (0.2)	1.6 (0.2)	1.7 (0.2)	0.017
BMI (kg/m^2^), mean (SD)		24.6 (4.8)	23.8 (4.2)	26.0 (5.4)	0.010
Comorbidities, *n* (%)					
Current or previous smoking		22 (16.4)	13 (15.3)	9 (18.4)	0.644
Diabetes mellitus		31 (23.1)	23 (27.1)	8 (16.3)	0.156
Hypertension		80 (59.7)	51 (60.0)	29 (59.2)	0.926
Hyperlipidaemia		83 (61.9)	53 (62.4)	30 (61.2)	0.897
Ischaemic heart disease		47 (35.1)	33 (38.8)	14 (28.6)	0.231
Previous heart failure		19 (14.2)	12 (14.1)	7 (14.3)	0.979
Atrial fibrillation		28 (20.9)	16 (18.8)	12 (24.5)	0.437
Previous stroke or TIA		11 (8.2)	6 (7.1)	5 (10.2)	0.530
Chronic kidney disease		56 (41.8)	55 (64.7)	1 (2.0)	<0.001
Treatment					
Medical therapy, *n* (%)	134				
Antiplatelets		49 (36.6)	35 (41.2)	14 (28.6)	0.145
Anticoagulation		21 (15.7)	13 (15.3)	8 (16.3)	0.874
Statins		74 (55.2)	47 (55.3)	27 (55.1)	0.983
Beta blockers		68 (50.7)	34 (40.0)	34 (69.4)	0.001
ACEi or ARB		30 (22.4)	17 (20.0)	13 (26.5)	0.382
AV replacement, n (%)	85	N/A	32 (37.6)	N/A	N/A
Duration to AV replacement	13	N/A	1.9 (1.7)	N/A	N/A
ICD implantation, *n* (%)	49	N/A	N/A	4 (8.2)	N/A
Outcomes					
Follow-up duration (years), mean (SD)	134	2.9 (2.1)	2.7 (2.2)	3.4 (1.8)	0.042
AMI outcomes, *n* (%)	49	N/A	N/A	5 (10.2)	N/A
Subsequent heart failure, *n* (%)	134	31 (23.1)	25 (29.4)	6 (12.2)	0.023
Stroke outcomes, *n* (%)		9 (6.7)	3 (3.5)	6 (12.2)	0.073
CV hospitalisation, *n* (%)		18 (13.4)	12 (14.1)	6 (12.2)	0.759
All-cause mortality, *n* (%)		42 (31.3)	35 (41.2)	7 (14.3)	0.001

Abbreviations: ACEi = angiotensin-converting enzyme inhibitor; AMI = acute myocardial infarction; ARB = angiotensin receptor blocker; AS = aortic stenosis; AV = aortic valve; BMI = body mass index; BSA = body surface area; CV = cardiovascular; HOCM = hypertrophic obstructive cardiomyopathy; ICD = implantable cardioverter defibrillator; N/A = not applicable; SD = standard deviation; TIA = transient ischaemic attack.

**Table 2:** Time-to-event analyses of outcomes in patients with left ventricular outflow tract obstruction.

Variables	All-Cause Mortality ^1^		Subsequent Heart Failure ^2^		Stroke Outcome ^2^		CV Hospitalisation ^2^	
	HR (95% CI)	*p* Value	HR (95% CI)	*p* Value	HR (95% CI)	*p* Value	HR (95% CI)	*p* Value
Univariate analysis								
HOCM Severe AS	Reference 3.54 (1.57–7.98)	<0.001	Reference 2.79 (1.21–6.45)	0.016	Reference 0.29 (0.07–1.16)	0.080	Reference 1.21 (0.46–3.20)	0.696
Multivariable analysis ^3^								
HOCM Severe AS	Reference 1.44 (0.48–4.07)	0.542	Reference 3.49 (1.37–8.94)	0.009	Reference 0.43 (0.05–3.61)	0.438	Reference 0.81 (0.15–4.42)	0.810

^1^ Cox proportional hazards regression model. ^2^ Fine and Gray competing risks model (for mortality). ^3^ Adjusted for age, female sex, BMI, chronic kidney disease, ischaemic heart disease and pressure gradient (AV mean pressure gradient for AS and LVOT gradient for HOCM). Abbreviations: AS = aortic stenosis; AV = aortic valve; BMI = body mass index; CI = confidence interval; CV = cardiovascular; HOCM = hypertrophic obstructive cardiomyopathy; HR = hazard ratio.

## Data Availability

The original contributions presented in this study are included in the article/Supplementary Materials.

## Supplementary Materials

The following supporting information can be downloaded at https://www.mdpi.com/article/10.3390/medicina61060971/s1. Figure S1. All-cause mortality in severe AS (with or without chronic kidney disease) and HOCM patients. Table S1: Echocardiographic parameters of severe AS and HOCM patients. Table S2: Multivariable time-to-event analyses of outcomes in patients with left ventricular outflow tract obstruction.
