# Supplementary material for: Longitudinal Outcomes of Left Ventricular Outflow Tract Obstruction in Aortic Stenosis Versus Hypertrophic Obstructive Cardiomyopathy
Source: Medicina (Kaunas). 2025 May 23;61(6):971. doi: 10.3390/medicina61060971 (PMC12195190; doi:10.3390/medicina61060971)
Supplement: Supplementary file 1 [file medicina-61-00971-s001.zip › medicina-3572427-supplementary.pdf]

Supplementary Table S1. Echocardiographic parameters of severe AS and HOCM patients

**Variables**	**Valid N**	**Overall** N = 134	**Severe AS** N = 85	**HOCM** N = 49	***p*-value**
AVA (cm^2), mean (SD)	92	0.8 (0.5)	0.7 (0.2)	2.2 (0.7)	**<0.001**
AVA index (cm^2/m^2), mean (SD)	92	0.5 (0.3)	0.4 (0.1)	1.3 (0.4)	**<0.001**
AV MPG (mmHg), mean (SD)	97	52.2 (19.0)	57.2 (13.9)	16.7 (10.4)	**<0.001**
Vmax (m/sec), mean (SD)	100	4.3 (1.0)	4.7 (0.5)	2.7 (1.0)	**<0.001**
AV PPG (mmHg), mean (SD)	103	80.1 (28.6)	89.9 (18.2)	34.1 (23.6)	**<0.001**
SV (ml), mean (SD)	132	62.3 (25.9)	68.6 (21.6)	51.2 (29.3)	**<0.001**
SV index (ml/m^2), mean (SD)	132	37.8 (14.7)	42.5 (12.1)	29.5 (15.1)	**<0.001**
DI, mean (SD)	97	0.2 (0.3)	0.2 (0.1)	0.6 (0.8)	**<0.001**
LVOT diameter (mm), mean (SD)	134	19.0 (6.0)	20.7 (1.9)	16.1 (8.9)	**<0.001**
LVOT VTI (mm), mean (SD)	119	24.6 (12.7)	22.0 (5.6)	30.9 (21.0)	**<0.001**

LVOT Vmax (m/s), mean (SD)	134	2.0 (1.5)	0.9 (0.2)	3.8 (0.9)	**<0.001**
LVOT gradient (mmHg), mean (SD)	134	24.2 (34.7)	3.5 (1.5)	60.1 (35.5)	**<0.001**
CO (L/min), mean (SD)	129	4.3 (1.9)	4.8 (1.6)	3.5 (2.2)	**<0.001**
CI (L/min/m^2), mean (SD)	129	2.6 (1.1)	3.0 (0.8)	2.0 (1.2)	**<0.001**
LVEF (%), mean (SD)	134	59.4 (13.8)	53.8 (13.7)	69.0 (7.0)	**<0.001**
LV mass (g), mean (SD)	133	250.1 (91.5)	232.5 (66.9)	280.3 (117.5)	**0.003**
LV mass index (g/m^2), mean (SD)	133	150.9 (45.9)	144.9 (40.0)	161.1 (53.5)	**0.048**
LVH, *n* (%)	134	103 (76.9)	65 (76.5)	38 (77.6)	0.982
RWT, mean (SD)	134	2.5 (5.6)	3.6 (6.9)	0.6 (0.2)	**0.003**
LVIDd (mm), mean (SD)	133	47.1 (7.6)	49.2 (6.8)	43.5 (7.7)	**<0.001**
LVIDs (mm), mean (SD)	133	30.5 (8.0)	33.0 (8.0)	26.3 (6.0)	**<0.001**
IVSd (mm), mean (SD)	134	13.0 (4.9)	10.3 (2.6)	17.5 (4.6)	**<0.001**

IVSs (mm), mean (SD)	133	18.0 (4.4)	16.2 (2.8)	21.1 (4.8)	**<0.001**
LVPWd (mm), mean (SD)	133	12.2 (2.5)	11.8 (2.1)	12.9 (2.9)	**0.008**
LVPWs (mm), mean (SD)	133	16.9 (3.4)	16.2 (2.6)	18.2 (4.2)	**<0.001**
LVEDV (ml), mean (SD)	133	106.7 (40.3)	116.9 (38.2)	89.2 (38.0)	**<0.001**
LVEDV index (ml/m^2), mean (SD)	133	64.5 (22.5)	72.2 (21.3)	51.4 (18.3)	**<0.001**
LVESV (ml), mean (SD)	134	40.3 (27.7)	47.7 (30.3)	27.5 (16.0)	**<0.001**
LVESV index (ml/m^2), mean (SD)	134	24.4 (16.5)	29.5 (17.9)	15.8 (8.3)	**<0.001**
EA, mean (SD)	128	1.0 (0.6)	1.0 (0.6)	0.8 (0.7)	0.101
Septal E/e’, mean (SD)	124	21.9 (13.0)	23.2 (15.4)	19.8 (7.3)	0.171
Lateral E/e’, mean (SD)	83	15.1 (7.7)	14.7 (8.3)	15.5 (7.3)	0.682
PASP (mmHg), mean (SD)	124	37.5 (13.6)	39.1 (15.3)	35.1 (10.2)	0.112
Moderate MR or worse, *n* (%)	103	10 (9.7)	3 (5.6)	7 (14.3)	0.187

Systolic anterior motion, *n* (%)	49	N/A	N/A	28 (57.1)	N/A

Abbreviations: AV – aortic valve; AVA – aortic valve area; CI – cardiac index; CO – cardiac output; DI – dimensionless index; IVS – interventricular septum; LV – left ventricular; LVEDV – left ventricular end diastolic volume; LVEF – left ventricular ejection fraction; LVESV – left ventricular end systolic volume; LVH – left ventricular hypertrophy; LVID – left ventricular internal diameter; LVOT – left ventricular outflow tract; LVPW – left ventricular posterior wall diameter; MPG – mean pressure gradient; MR – mitral regurgitation; PASP – pulmonary artery systolic pressure; PPG – peak pressure gradient; RWT – relative wall thickness; SD – standard deviation; SV – stroke volume; Vmax – peak aortic jet velocity; VTI – velocity-time integral

Supplementary Table S2. Multivariable time-to-event analyses of outcomes in patients with left ventricular outflow tract obstruction

**Variables**	**All-Cause Mortality[1]**		**HF Outcomes[2]**		**Stroke Outcomes[2]**		**CV Rehospitalisation[2]**	
	aHR (95% CI)[1]	*p*-value	aHR (95% CI)[1]	*p*-value	aHR (95% CI)[1]	*p*-value	aHR (95% CI)[1]	*p*-value
Age (years)	1.01 (0.98 to 1.04)	0.524	1.02 (0.98 to 1.06)	0.287	1.05 (0.96 to 1.14)	0.271	1.04 (0.98 to 1.10)	0.240
Female sex	1.66 (0.88 to 3.16)	0.116	1.04 (0.53 to 2.05)	0.905	1.23 (0.20 to 7.44)	0.819	2.13 (0.75 to 6.04)	0.154
BMI (per kg/m^2)	**0.90 (0.82 to 0.98)**	**0.011**	1.00 (0.92 to 1.09)	0.936	1.01 (0.89 to 1.15)	0.861	1.00 (0.88 to 1.15)	0.950
Chronic kidney disease	**3.08 (1.23 to 7.70)**	**0.010**	0.64 (0.25 to 1.66)	0.360	0.49 (0.05 to 4.60)	0.534	1.81 (0.35 to 9.28)	0.476

Ischaemic heart disease	1.59 (0.83 to 3.06)	0.168	1.22 (0.59 to 2.52)	0.593	0.20 (0.03 to 1.42)	0.107	**3.48 (1.35 to 8.97)**	**0.010**
Severe AS (vs. HOCM)	1.40 (0.48 to 4.07)	0.542	**3.49 (1.37 to 8.94)**	**0.009**	0.43 (0.05 to 3.61)	0.438	0.81 (0.15 to 4.42)	0.810
Pressure gradient[3] (per mmHg)	1.00 (0.98 to 1.02)	0.983	1.01 (1.00 to 1.02)	0.243	1.00 (0.99 to 1.01)	0.490	**1.01 (1.00 to 1.02)**	**0.011**

[1]Cox proportional hazards regression model

[2]Fine & Gray competing risks model (for mortality)

[3]Aortic valve mean pressure gradient for severe AS, LVOT gradient for HOCM

Abbreviations: aHR – adjusted hazard ratio; AS – aortic stenosis; AV – aortic valve; BMI – body mass index; CI – confidence interval; CV – cardiovascular; HOCM – hypertrophic obstructive cardiomyopathy

Supplementary Figure S1. All-cause mortality in severe AS (with or without chronic kidney disease) and HOCM

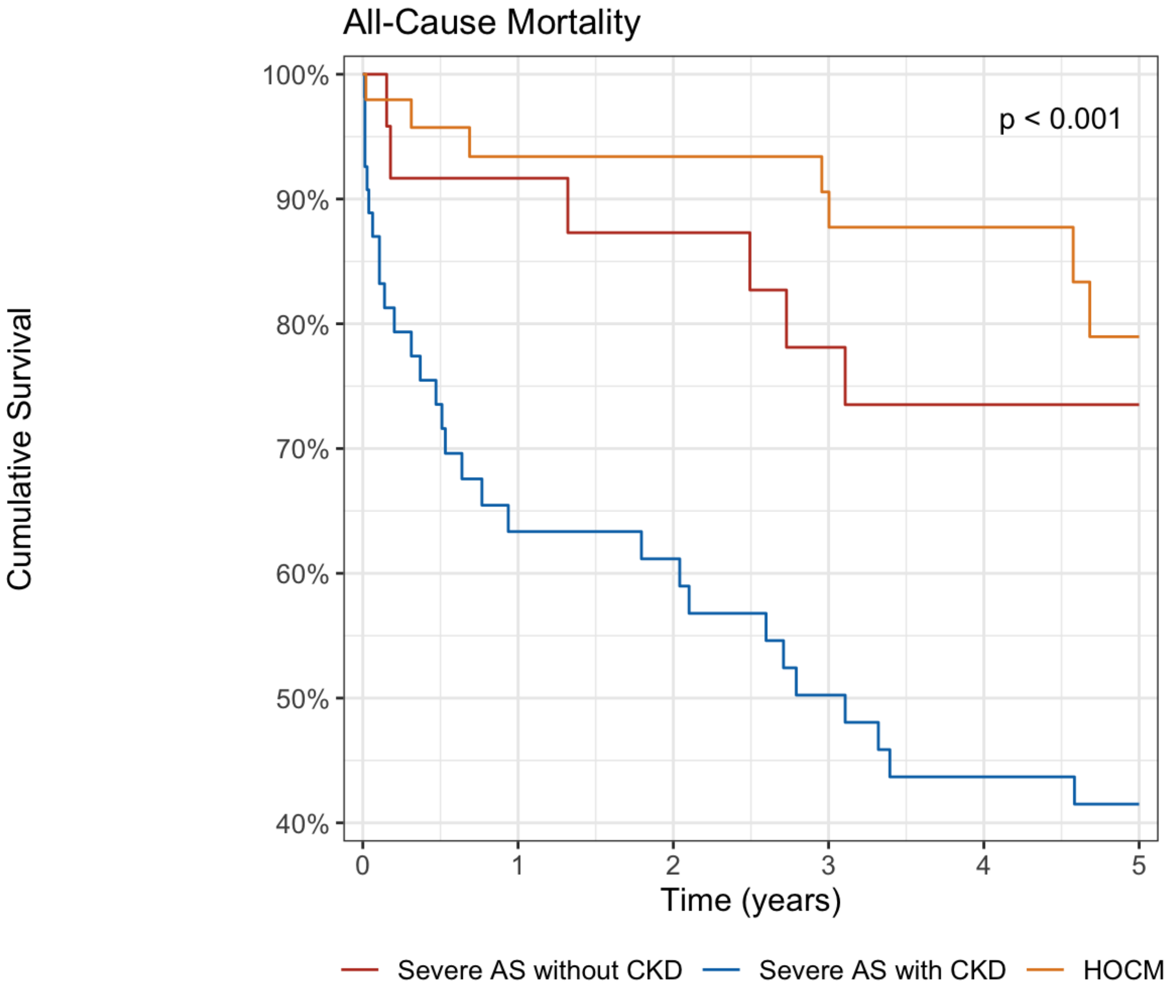

At Risk

Severe AS without CKD	30	22	20	17	16	16
Severe AS with CKD	55	30	28	23	20	18
HOCM	49	40	38	32	26	17
